# Abnormal expression and clinical value analysis of long noncoding RNA cancer susceptibility candidate 2 in children with severe pneumonia complicated with respiratory failure

**DOI:** 10.1111/crj.13510

**Published:** 2022-06-03

**Authors:** Jie Ni, Junfei Lu, Ding Lu

**Affiliations:** ^1^ Pediatrics Shanghai Eighth People's Hospital Shanghai China; ^2^ Engineering Department East China University of Science and Technology Shanghai China

**Keywords:** CASC2, respiratory failure, severe pneumonia, survival rate

## Abstract

**Objective:**

Severe pneumonia occurs commonly in children and is the main cause of clinical infant mortality. This study tested the expression pattern of long noncoding RNA cancer susceptibility candidate 2 (CASC2) in the serum of children with severe pneumonia and explored its clinical values.

**Methods:**

Serum levels of CASC2 were detected in 145 children with severe pneumonia. All cases were divided into two groups based on their respiratory failure (RF) condition. Receiver operating characteristic (ROC) and Kaplan–Meier (K‐M) curves were plotted for the diagnostic and prognostic ability evaluation. Multivariate cox regression analysis was done for the examination of independent influence factors.

**Results:**

The serum levels of CASC2 significantly decreased in children with severe pneumonia in contrast with healthy individuals and reached the lowest value in those with RF. Serum CASC2 can distinguish severe pneumonia and predicted the development of RF. Based on the 28‐day survival data, cases with low CASC2 levels had a poor survival rate. CASC2 (hazard ratio [HR] = 0.068, 95% confidence interval [CI] = 0.016–0.292, *P* < 0.001) and age (HR = 2.806, 95% CI = 1.240–6.394, *P* < 0.001) were independent influence factor for the poor prognosis of children with severe pneumonia.

**Conclusion:**

Downregulation of serum CASC2 was related to the occurrence of RF in children with severe pneumonia and may be a predictor of the poor prognosis. This study will provide a potential biomarker for severe pneumonia treatment in clinic.

## Graphical abstract

Serum levels of CASC2 significantly decreased in children with severe pneumonia. Serum CASC2 can distinguish severe pneumonia, and predicted the development of respiratory failure. Downregulation of serum CASC2 may be a predictor of the poor prognosis.

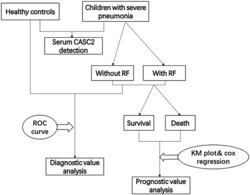

## 1 INTRODUCTION

Community‐acquired pneumonia (CAP) is caused outside the hospital by a variety of microorganisms including bacteria, viruses, chlamydia, and mycoplasma.^1^ Children's immune system is not mature, prone to pathogen infection, and eventually developed into pneumonia.^2^ Severe pneumonia occurs when the child has severe ventilation dysfunction or internal and external pulmonary complications. According to the previous report, approximately 6.6%–16.7% of children develop severe pneumonia, which is the main cause of clinical infant mortality.^3^ Respiratory failure (RF) is a clinical syndrome of physiological and metabolic disorders caused by pulmonary ventilation or ventilation dysfunction.^4^ It can be used as a diagnostic criterion for severe pneumonia. The imbalance of pro‐inflammatory and anti‐inflammatory responses is the key to the occurrence of severe pneumonia in children.^5^ Therefore, accurate monitoring of the disease and prognosis is of great significance in blocking the inflammatory cascade reaction and alleviating symptoms.

Cancer susceptibility candidate 2 (CASC2) is a long non‐coding RNA, which is located on human chromosome 10; the dysregulation of CASC2 is firstly detected in patients with endometrial cancer.^6^ In recent years, CASC2 is reported to be aberrantly expressed in a variety of tumors, such as hepatocellular carcinoma, papillary thyroid cancer, etc.^7^, ^8^ Notably, CASC2 has been highlighted for its role in the onset and progress of various human lung diseases.^9^, ^10^ The downregulation of CASC2 has been demonstrated to promote lung adenocarcinoma and shows a negative correlation with the development of drug resistance of the tumor patients.^9^, ^11^ Downregulation of CASC2 is detected in both sepsis patients and LPS‐treated HPAEpiC, contributing to the cell inflammatory and oxidative damages.^10^, ^12^ It was also shown that overexpression of CASC2 can alleviate lung damage in newborns caused by bronchopulmonary dysplasia.^13^ These evidences spurred our interest in characterizing CASC2 as a diagnostic and prognostic marker in CAP.

Herein, we detected the expression pattern of CASC2 in the serum of children with severe pneumonia and explored its clinical values. In addition, the correlation between CASC2 and prognosis of patients was also examined.

## 2 MATERIALS AND METHODS

### 2.1 The recruitment of study subjects

One hundred forty‐five children with severe pneumonia admitted to the intensive care unit (ICU) of Shanghai Eighth People's Hospital from April 2020 to June 2021 were selected. All cases met the diagnostic criteria of the British Thoracic Society 2011 guidelines for the management of severe pneumonia in children.^14^ The RF condition of all patients was recorded, and the diagnostic criteria of RF were the PaO2 level less than 50 mm Hg or a need of mechanical ventilation. Exclusion criteria are as follows: (1) combined with congenital diseases, such as congenital heart disease, abnormal laryngeal development, trachea or lung dysplasia, etc.; (2) suffering from bronchial foreign body tuberculosis infection and other diseases; (3) diseases of the blood system and immune system; (4) suffering from severe dysfunction of important organs of malignant tumor; (5) prior to admission, they had received drug treatment such as hormone immunotherapy; (6) suffering from serious mental illness; (7) aged less than 28 days or more than 14 years old.

### 2.2 Obvervational index

Venous blood was collected from each subject within 24 h of admission. SysemxXE‐5000 hematology analyzer was used for a routine blood test and white blood cell count (WBC) was determined. The levels of C‐reactive protein (CRP) and procalcitonin (PCT) were determined by immunofluorescence quantitative assay. Enzyme‐linked immunosorbent assay (ELISA) was performed for the measurement of the levels of D‐dimer and interleukin‐6 (IL‐6).

### 2.3 Clinical outcome

All children with severe pneumonia were followed for 28 days, and their survival was recorded.

### 2.4 Serum CASC2‐level detection via RT‐qPCR

Two microliter venous blood was collected from each subject and kept at 25°C for 30 min. The serum samples were collected by centrifugation at 2000×*g* for 15 min at 4°C. Total RNA was extracted from serum samples using Trizol kit (Pufa, Shanghai, China). The total RNA extracted was reverse‐transcribed with reference to PrimeScript™ RT reagent Kit (TaKaRa, Dalian, China). TB Green™ Fast qPCR Mix was purchased from TaKaRa (Dalian, China) and used for the qualitative reverse transcription polymerase chain reaction (qRT‐PCR) assay. PCR conditions: 95°C for 15 min; 95°C for 10 s; 60°C for 20 s; a total of 40 cycles. Three parallel samples were set for each sample, and the average value of the three samples was taken. The expression level of lncRNA CASC2 was calculated by 2^−ΔΔCt^ method.

### 2.5 Statistical analysis

SPSS 22.0 software was used for statistical analysis. Measurement data were expressed as mean ± standard deviation (SD) and compared between groups using one‐way analysis of variance (ANOVA). The counting data were expressed as numbers and percentage and compared between groups using the chi‐square test. The receiver operating characteristic (ROC) curve was plotted, and the diagnostic value was evaluated via calculating sensitivity and specificity. The Kaplan–Meier (K‐M) plot was established for the survival analysis with the log‐rank test. *P* < 0.05 indicated a significant difference.

## 3 RESULTS

### 3.1 Clinical data comparison for CAP children with or without RF

As shown in Table 1, the clinical data of all recruited children were summarized and compared. Sixty‐eight healthy individuals were included, with a mean age of 3.63 ± 1.42, including 35 males and 32 females. A total of 145 children with severe pneumonia were recruited, and they were divided into two groups according to the RF condition. For children with or without RF, the mean age was 3.62 ± 1.51 and 3.11 ± 1.47, respectively. Both age and gender exhibited no significant difference among healthy, without RF and with RF groups (all *P* > 0.05). For children without RF, 3.33% of cases showed positive blood bacterial culture, and 31.67% exhibited positive sputum bacterial culture. For cases with RF, the blood or sputum‐positive bacterial culture rates were 4.71% and 42.4%, respectively. And the distribution exhibited no significant difference in comparison with those without RF (*P* > 0.05).

**TABLE 1:** Clinical data comparison for CAP children with or without respiratory failure (RF)

Items	Healthy individuals (*n* = 68)	Severe pneumonia	
		Without RF (*n* = 60)	With RF (*n* = 85)
Age, year	3.63 ± 1.42	3.62 ± 1.51	3.11 ± 1.47^#^
Sex, male/female	35/32	41/19	50/35
Antibiotic use, *n* (%)	‐	48 (80.00)	69 (81.18)
Positive bacterial culture (blood), *n* (%)	‐	2 (3.33)	4 (4.71)
Positive bacterial culture (sputum), *n* (%)	‐	19 (31.67)	36 (42.4)
Laboratory data			
CRP, mg/ml	4.08 ± 1.38	17.62 ± 9.99^***^	22.90 ± 12.48^***^ ^,^ ^##^
IL‐6, pg/ml	3.63 ± 1.50	61.35 ± 7.58^***^	98.72 ± 26.28^***^ ^,^ ^###^
PCT, ng/ml	0.21 ± 0.10	0.31 ± 0.13^***^	0.35 ± 0.21^***^
WBC, 10^9^/L	8.02 ± 1.77	9.85 ± 2.04^***^	10.37 ± 1.78^***^
D‐dimer, μg/ml	0.24 ± 0.10	0.74 ± 0.155^***^	1.91 ± 0.86^***^ ^,^ ^###^

*Note*: Data are expressed as number or mean ± standard deviation.

Abbreviations: CRP, C‐reactive protein; IL‐6, interleukin 6; PCT, procalcitonin; RF, respiratory failure; WBC, white blood cell count.

^***^ *P* < 0.001, compared with healthy individuals.

^#^ *P* < 0.05compared with children without RF.

^##^ *P* < 0.01, compared with children without RF.

^###^ *P* < 0.001, compared with children without RF.

The laboratory data of all study subjects were also recorded. As shown in Table 1, levels of CRP, IL‐6, and D‐dimmer showed a progressively increasing trend in healthy individuals, children without RF and with RF groups. Those levels approach the peak level in children with RF, and the difference reached a significant level (*P* < 0.05). A similar increasing trend was also observed in PCT and WBC, and the levels of the two indicators were higher in children with severe pneumonia than healthy individuals, but the difference was not significant between children with RF and without RF (*P* > 0.05).

### 3.2 Serum CASC2‐level analysis in children with severe pneumonia

**FIGURE 1:**
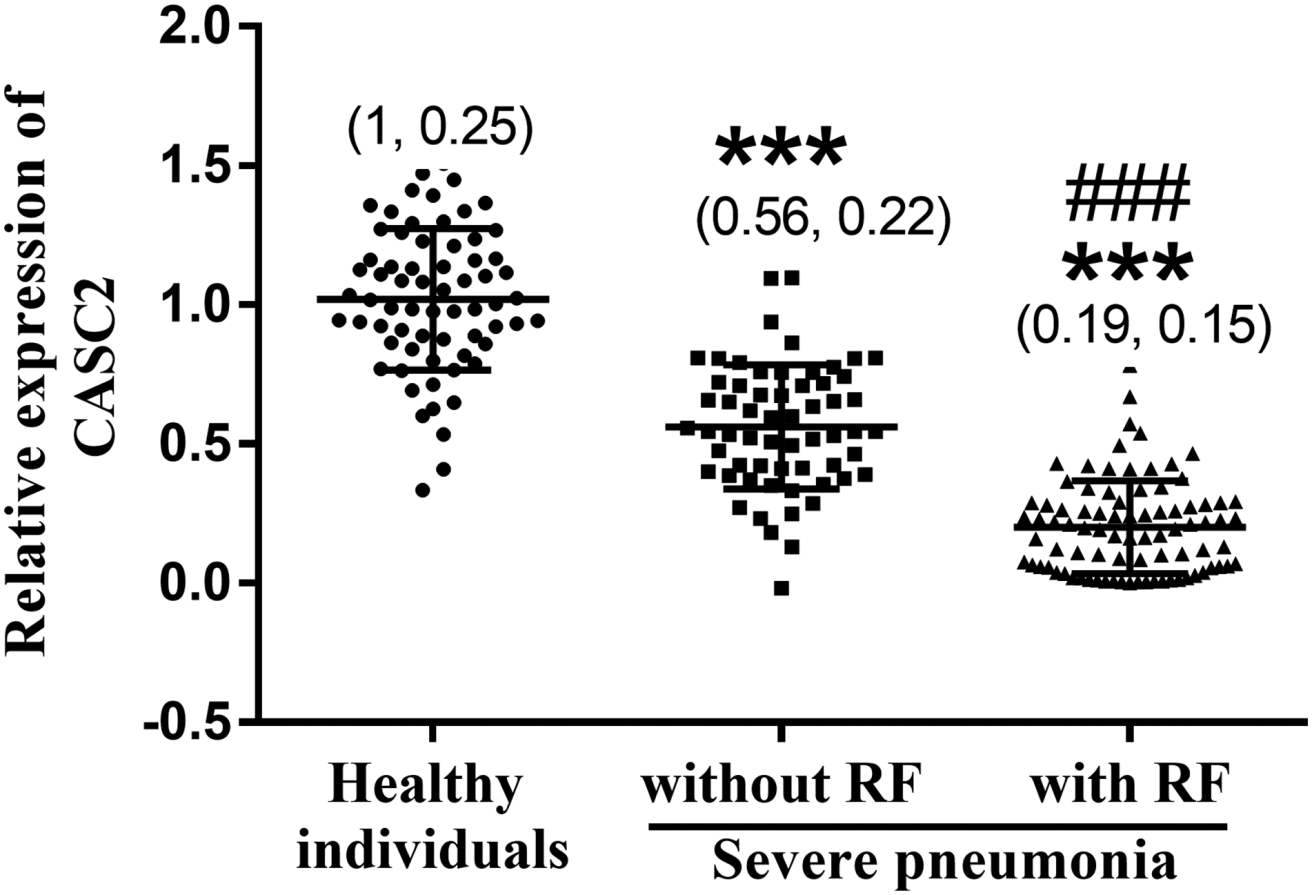
Serum cancer susceptibility candidate 2 (CASC2) levels analysis in children with severe pneumonia. CASC2 significantly decreased in children with severe pneumonia in contrast with healthy individuals and reached the lowest value in children with RF. *** means *P* less than 0.001 in comparison with healthy individuals; ### means *P* less than 0.001 in comparison with children with respiratory failure (RF). Data were expressed as (mean values and standard deviation [error bar])

qRT‐PCR was applied for mRNA level detection. As shown in Figure 1, the serum levels of CASC2 significantly decreased in children with severe pneumonia in contrast with healthy individuals (*P* < 0.001) and reached the lowest value in children with RF (*P* < 0.001).

### 3.3 Diagnostic value analysis of serum CASC2 for severe pneumonia

**FIGURE 2:**
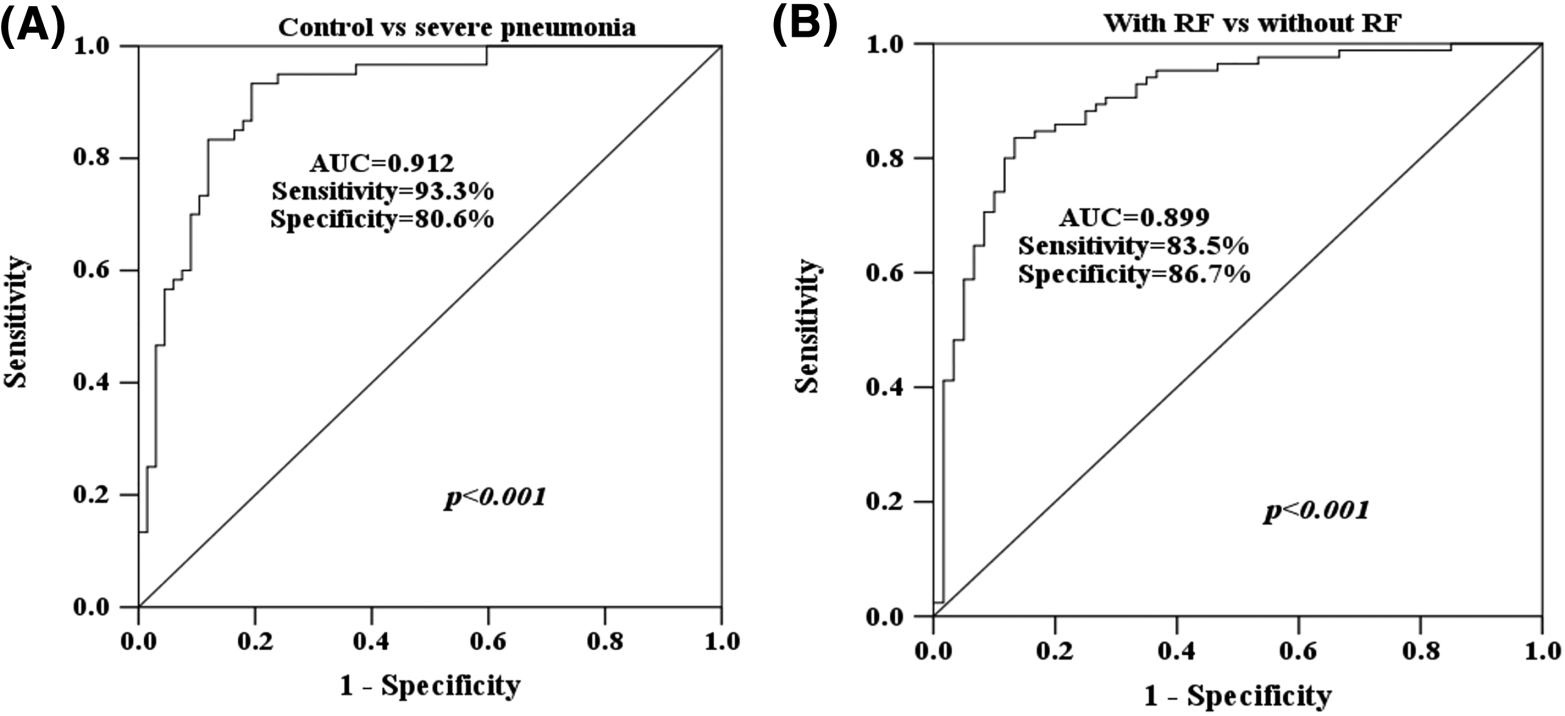
Diagnostic value analysis of serum cancer susceptibility candidate 2 (CASC2) for severe pneumonia. (A) Diagnostic value of CASC2 in distinguish severe pneumonia from healthy people. (B) Diagnostic value of CASC2 in distinguish children with respiratory failure (RF) from those without RF

Based on the serum levels of CASC2 in healthy individuals and children with severe pneumonia, the ROC curve was established. As shown in Figure 2A, the ROC curve indicated the diagnostic value of CASC2 for severe pneumonia from healthy people with the ROC of 0.912; the sensitivity and specificity were 93.3% and 80.6%, respectively. Furthermore, the resolving power of CASC2 between severe pneumonia with and without RF was evaluated. As Figure 2B revealed, serum CASC2 can distinguish children with RF from those without RF (area under the curve [AUC] = 0.899; sensitivity = 83.5%, specificity = 86.7%).

### 3.4 Correlation between lncRNA CASC2 and clinical indicators

Pearson's correlation analysis was done to evaluate the correlation between CASC2 and clinical indicators. As shown in Table 2, a positive association was detected between CASC2 and age (*r* = 0.212, *P* = 0.010). In addition, serum CASC2 was negatively correlated with the levels of CRP (*r* = −0.336, *P* < 0.001), IL‐6 (*r* = −0.654, *P* < 0.001), and D‐dimer (*r* = −0.504, *P* < 0.001).

**TABLE 2:** Correlation between lncRNA cancer susceptibility candidate 2 (CASC2) and clinical indicators

Items	Correlation (*r*)	*P* value
Age, year	0.212	0.010
Sex, male/female	−0.067	0.423
CRP, mg/ml	−0.336	<0.001
IL‐6, pg/ml	−0.654	<0.001
PCT, ng/ml	−0.149	0.074
WBC, 10^9^/L	−0.044	0.599
D‐dimer, μg/ml	−0.504	<0.001

Abbreviations: CRP, C‐reactive protein; IL‐6, interleukin 6; PCT, procalcitonin; WBC, white blood cell count.

### 3.5 Serum CASC2 can predict the clinical outcome of children with severe pneumonia

**FIGURE 3:**
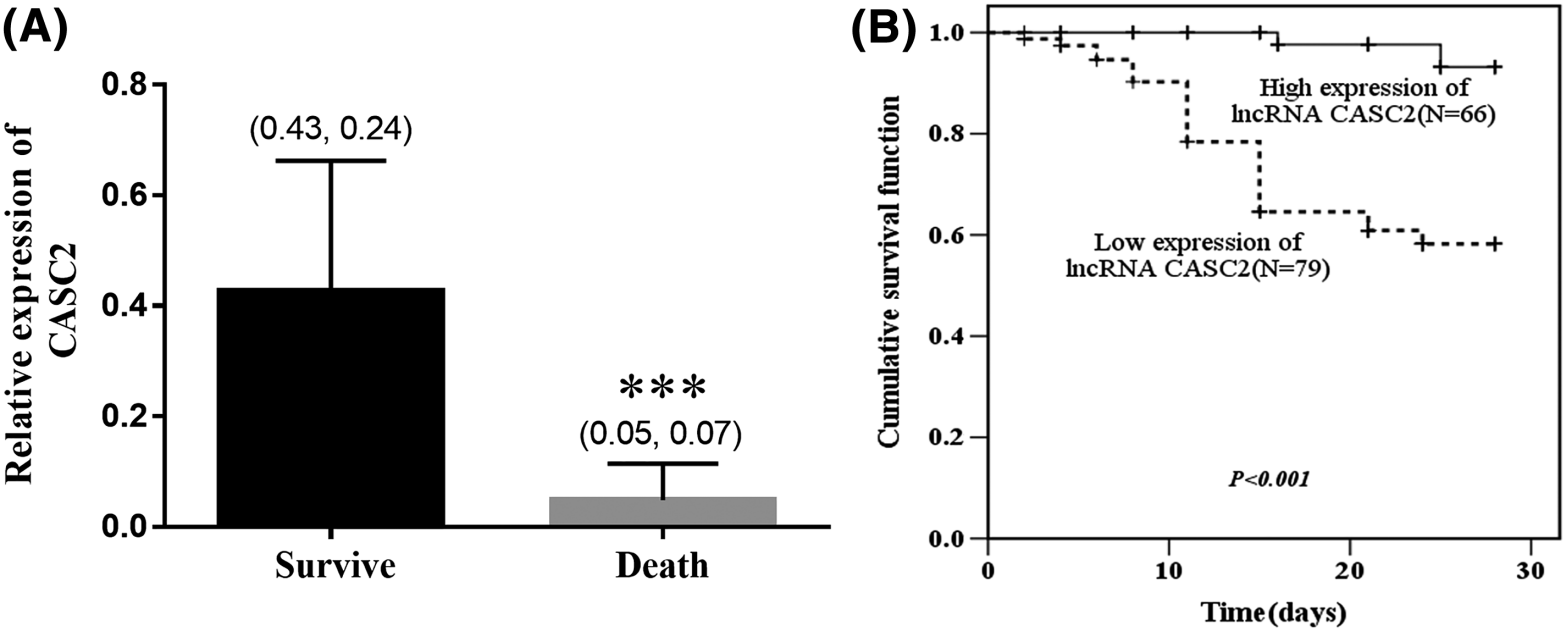
Predictive value of serum respiratory failure (CASC2) for clinical outcome of severe pneumonia children. (A) Children in death group owned a remarkably low level of CASC2 compared the survivors. *** means *P* less than 0.001 in comparison with survivors. Data were expressed as (mean values and standard deviation [error bar]). (B) Kaplan–Meier (K‐M) plot based on the 28‐day survival data of all children with severe pneumonia. Cases with low CASC2 levels had poor survival rate (long rank *P* < 0.001)

Based on the 28‐day survival data, a total of 116 (80.0%) children survived. Levels of CASC2 were compared in survival cases and death ones. As shown in Figure 3A, children in death gorup owned a remarkably low level of CASC2 compared the survivors (*P* < 0.001). According to the mean value of serum CASC2 in all children with severe pneumonia, all cases were divided into low CASC2 group (*n* = 79) and high CASC2 group (*n* = 66). It can be seen that cases with low CASC2 levels had a poor survival rate (*P* < 0.001, Figure 3B). Furthermore, the multivariate cox regression analysis demonstrated the independent influence of CASC2 value (>0.35 vs. <0.35; hazard ratio [HR] = 0.068, 95% confidence interval [CI] = 0.016–0.292, *P* < 0.001) and age (<3.3 years old [mean value] vs. >3.3 years old; HR = 2.806, 95% CI = 1.240–6.394, *P* < 0.001) on the poor prognosis of children with severe pneumonia (Table 3).

**TABLE 3:** Multivariate cox regression analysis of factors affecting the prognosis of CAP children

Items	HR	95% CI	*P* value
Age, year	2.806	1.240–6.349	0.013
Sex, male/female	1.056	0.469–2.380	0.895
CRP, mg/ml	2.015	0.839–4.843	0.117
IL‐6, pg/ml	2.234	0.924–5.406	0.075
PCT, ng/ml	1.926	0.803–4.624	0.142
WBC, 10^9^/L	1.787	0.819–3.900	0.145
D‐dimer, μg/ml	1.575	0.727–3.412	0.249
CASC2	0.068	0.016–0.292	<0.001

Abbreviations: CI, confidence interval; CRP, C‐reactive protein; HR, hazard ratio; IL‐6, interleukin 6; PCT, procalcitonin; WBC, white blood cell count.

## 4 DISCUSSION

Pneumonia is a common respiratory disease, especially in infants and young children. The morbidity and mortality of pneumonia both rank first among children's diseases.^15^ Without timely and effective diagnosis and treatment, pneumonia can easily develop into severe pneumonia accompanied with serious complications, such as RF, seriously threatening children's health and life safety.^16^ LncRNA CASC2 is closely related to the occurrence and development of various human lung diseases. It has been reported that CASC2 can inhibit the progression of lung adenocarcinoma and is associated with drug resistance in cancer patients.^9^, ^11^ In addition, CASC2 is also involved in the regulation of LPS‐induced lung injury; overexpression of CASC2 can attenuate lung damage in newborns caused by bronchopulmonary dysplasia.^10^, ^12^, ^13^ In the current study, a reduction of serum CASC2 was detected in the serum of children with severe pneumonia in comparison with healthy individuals. And its diagnostic ability for severe pneumonia was indicated by the ROC curve.

RF is a severe complication of severe pneumonia, which is the main contributor of the disease‐related deaths.^17^ In the current study, a total of 145 children with severe pneumonia were recruited. As reported, approximately 58%–87% of CAP patients can develop into RF, which is considered to be the main cause of death.^18^ In the current study, the occurrence rate of RF in all children with severe pneumonia was 59%, which was consistent with the previous evidence. Levels of CASC2 in children with or without RF were further compared, and the drop‐off of serum CASC2 was detected in children with RF. Besides, ROC curve indicated that serum CASC2 can distinguish children with RF from those without RF. The findings reflected that serum CASC2 was related to the RF progression of children with severe pneumonia.

On the basis of the blood biochemical index, children with severe pneumonia exhibited high levels of CRP, IL‐6, PCT, and D‐dimer. With the occurrence of inflammatory injury, serum CRP levels can be significantly increased.^19^ In the present study, the increased CRP level was detected in children with severe pneumonia. And for cases that underwent RF, CRP levels worsened. It reflected the inflammatory injury for severe pneumonia children, especially for those with RF. Similarly, an increasing trend of IL‐6 was also detected in children with severe pneumonia. As previously reported, levels of IL‐6 peak within hours after bacterial infection, especially in severe cases.^20^ The significant elevation of IL‐6 has been shown to predict the onset of RF.^21^ Consistently, a significant increase of IL‐6 was detected in children with RF. Another indicator of bacterial inflammatory disease infection, PCT, also showed higher levels in children with severe pneumonia.^22^ Although PCT levels increased in children with RF in comparison with those without RF, the difference did not reach a significant level. D‐dimer is a specific fibrin degradation product, which can be used as a molecular marker to diagnose diffuse intravascular coagulation and reflect thrombin production and fibrinolytic activity.^23^ In severe pneumonia, D‐dimer level is considered to be a useful biomarker to assess the disease severity.^24^ According to the present findings, increased D‐dimer was detected in children with RF compared with those without RF, reflecting the predictive value of D‐dimer in the severity of severe pneumonia. Interestingly, decreased CASC2 levels showed a negative correlation with the levels of CRP, IL‐6, PCT, and D‐dimer in children with severe pneumonia. The close association supported our speculation about the important role of CASC2 in the clinical outcome of children with severe pneumonia. In the current study, we focus on the mRNA pattern of CASC2 in pneumonia patients; its genotype data at the gene level was not included. In addition, measurement of CASC2 in the children who had pneumonia after they had recovered was not included, which should be elucidated in future study.

As a previous study reported, nearly 20% of children with severe pneumonia are at risk of treatment failure.^25^ In the current study, nearly 20% of cases died from severe pneumonia during the 28‐day follow‐up, and they had low levels of serum CASC2. Furthermore, the K‐M plot indicated that cases with low CASC2 levels had a poor survival rate, and CASC2 can independently influence the survival rate of children with severe pneumonia. As a previous study reported, CASC2 downregulation contributes the lung cell inflammatory and oxidative damages and further aggravates sepsis‐induced lung injury.^10^, ^12^ On the basis of the findings, we speculated that the independent influence role of serum CASC2 on the poor prognosis might be related to its regulatory role in cell inflammatory and oxidative damages. Besides, analysis of prognostic factors in this study showed that age was an independent risk factor affecting the prognosis of children with severe pneumonia. The cure rate of younger children was significantly lower than that of older children. It is considered that it may be related to immature respiratory tract anatomical structure, weak cough reflex, respiratory mucosal barrier, and poor autoimmune ability.^26^ Therefore, more attention should be paid to younger children; timely identification of pathogenic factors and appropriate symptomatic treatment should be actively carried out to improve the cure rate of children.

In conclusion, downregulation of serum CASC2 was related to the occurrence of RF in children with severe pneumonia and may be a predictor of clinical outcomes. This study will provide a potential biomarker for severe pneumonia treatment in clinic. Attention should be paid to the changes of serum CASC2 levels in children with severe pneumonia complicated with RF, and timely intervention should be taken to improve the prognosis. But the predicting role of CASC2 in pneumonia should be confirmed in another population.

## CONFLICT OF INTEREST

There are no conflicts of interest.

## AUTHOR CONTRIBUTIONS

Jie Ni designed the study. Junfei Lu conducted the experiment and analyzed the data. Ding Lu wrote the manuscript. All authors have agreed to the publication of this study.

## ETHICS STATEMENT

This study was approved by the Ethics Committee of Shanghai Eighth People's Hospital. All the legal guardians of included patients and healthy controls signed written informed consent.

## Data Availability

Data for this study can be obtained by contacting the corresponding author.
